# ‘One man’s medicine is another man’s poison’: a qualitative study of user perspectives on low intensity interventions for Obsessive-Compulsive Disorder (OCD)

**DOI:** 10.1186/s12913-016-1433-3

**Published:** 2016-05-18

**Authors:** Jasmin Knopp-Hoffer, Sarah Knowles, Peter Bower, Karina Lovell, Penny E. Bee

**Affiliations:** NIHR School for Primary Care Research, Manchester Academic Health Science Centre, University of Manchester, Manchester, UK; School of Nursing, Midwifery and Social Work, The University of Manchester, Manchester, UK

**Keywords:** Obsessive-compulsive disorder, Low intensity intervention, Acceptability, Therapy uptake, Therapy engagement

## Abstract

**Background:**

Low intensity interventions based on cognitive-behavioral therapy (CBT) such as computerized therapy or guided self-help can offer effective and accessible care for mild to moderate mental health problems. However, critics argue that by reducing therapist input and the level of experience of the professionals delivering therapy, low intensity interventions deprive users of critical ‘active ingredients’. Thus, while demand management arguments support the use of low intensity interventions for OCD, their integration into existing mental health services remains incomplete. Studies of user views of low intensity interventions can offer valuable insights to define their role and optimize their implementation in practice.

**Methods:**

Qualitative interviews (*n* = 36) in adults with OCD explored user perspectives on the initiation, continuation and acceptability of two low intensity CBT interventions: guided self-help (6 h of professional support) and computerized CBT (1 h of professional support), delivered within the context of a large pragmatic effectiveness trial (ISRCTN73535163).

**Results:**

While uptake was relatively high, continued engagement with the low intensity interventions was complex, with the perceived limitations of self-help materials impacting on users’ willingness to continue therapy. The addition of professional support provided an acceptable compromise between the relative benefits of self-help and the need for professional input. However, individual differences were evident in the extent to which this compromise was considered necessary and acceptable. The need for some professional contact to manage expectations and personalize therapy materials was amplified in users with OCD, given the unique features of the disorder. However, individual differences were again evident regarding the perceived value of face-to-face support.

**Conclusions:**

Overall the findings demonstrate the need for flexibility in the provision of low intensity interventions for OCD, responsive to user preferences, as these preferences impact directly on engagement with therapy and perceptions of effectiveness.

## Background

Psychological therapies, especially those based on cognitive-behavioral principles, remain critical to high quality mental health care but their delivery is undergoing major transformation [1]. Historically, due to skill shortages and the resources needed to deliver individual, face-to-face therapy, demand for individual psychotherapy has outstripped supply. This has resulted in poor access and long waiting lists [2]. Increasingly, the focus is shifting towards the development and implementation of more accessible and cost-effective approaches to therapy [3].

Within the UK, mental health services for common mental health problems are delivered according to a stepped care model. This aims to optimize effectiveness and increase access by directing users to the most appropriate and least resource-intensive care. Higher intensity services, delivered by more experienced therapists, are reserved for those who are judged unlikely to benefit from low intensity treatments or those who have already failed to do so.

Therapist-delivered CBT, including exposure and response prevention is the mainstay of psychological treatment for OCD [4]. Within treatment guidelines, a distinction is drawn between high intensity interventions (i.e., CBT with exposure and response prevention requiring more than 10 h of direct therapist-client contact) and low intensity interventions (requiring ten hours or less, delivered by less experienced therapists – called psychological wellbeing practitioners). ‘Low intensity’ interventions are recommended treatment options at lower levels of the stepped care model and make use of ‘health technologies’ (e.g., computerized CBT or self-help manuals) to convey some or all of the therapeutic content [5].

The drive to deliver more efficient services occurs in the context of a broader shift towards improving quality of care, including greater opportunities for patient preference and choice [6]. Requiring users to attend regular face-to-face therapy may not be suitable for all [7]. High transportation costs and rural living, competing social and occupational commitments, physical or psychological impairment and stigma are all potential factors, which may discourage attendance [8–11].

Despite the potential advantages of low intensity interventions, studies highlight significant dropout rates, often exceeding those in high intensity therapies [12–15]. Poor engagement with low intensity interventions may reflect reduced contact with a professional. The therapist-client relationship has long been considered critical in encouraging engagement [16], individualizing care [17] and meeting user expectations regarding the legitimacy and quality of psychological therapy [18]. Research in individuals with depression and anxiety [19–22] has identified both perceived advantages and disadvantages of low intensity interventions, highlighting individual differences in the value placed on professional support. However, depression and anxiety differ significantly from OCD.

OCD is a unique and debilitating mental health condition, with an estimated lifetime prevalence of 2–3 % [23], characterized by a heterogeneous pattern of repetitive, intrusive and anxiety-provoking thoughts (the obsessions) and behaviors and/or mental rituals (the compulsions) aimed at reducing this anxiety [24]. OCD is associated with reduced quality of life and substantial role impairment [25], and without treatment will follow a chronic course [26]. Yet, help seeking is commonly delayed for up to ten years [27]. Certain symptoms (in particular intrusive egodystonic thoughts of a sexual, religious or violent nature) have been associated with a reduced likelihood of disclosure [28], greater stigma [29, 30] and an increased need for individualized care [5]. These factors, and their possible conflicting implications for treatment preferences, render the acceptability of low intensity interventions for OCD uncertain. Understanding user experience is vital to determine their role in psychological services for OCD and the most appropriate method by which to optimize user benefit and system efficiency.

## Methods

This paper discusses the analysis of qualitative data from 36 adults with a clinical diagnosis of OCD who participated in individual interviews to assess the acceptability, uptake and engagement with guided self-help and computerized CBT (cCBT). This study formed part of a randomized controlled trial (RCT; *OCTET*) comparing the clinical- and cost-effectiveness of these interventions while waiting for high intensity face-to-face CBT to remaining on the waiting list for high intensity therapy, in adults with OCD [31]. Participant recruitment took place between 2012 and 2014.

Participants were primarily accessed through existing waiting lists of stepped care mental health services – called ‘Increasing Access to Psychological Therapies’ in the United Kingdom (IAPT). An OCD diagnosis was confirmed through the Mini International Neuropsychiatric Interview (M.I.N.I.) [32]. Adults (≥18 years) with an OCD diagnosis (M.I.N.I.) and clinically relevant symptoms of OCD, as defined by a self-rated Yale-Brown Obsessive Compulsive Scale (Y-BOCS; [33, 34]) score of ≥16, were eligible for inclusion. Exclusion criteria were current psychological treatment for OCD, organic brain disease, current symptoms of psychosis, alcohol or substance dependence, active suicidal ideation and literacy or language difficulties, which may have precluded participation.

The trial enabled us to explore the direct comparison between delivery formats and the level, mode and content of professional support. Participants in both the guided self-help- and cCBT arms, received some support from a psychological wellbeing practitioner, a specially trained professional who provides low intensity interventions within IAPT services [35]. Psychological wellbeing practitioners were trained in the delivery of face-to-face CBT but had varied expertise in supporting the use of cCBT and guided self-help. All received specific training as part of the trial.

*Guided Self-Help* consisted of a self-help book (focused on exposure and response prevention) ‘Overcoming OCD: a workbook’ written by the trial team. Participants received weekly guidance from a psychological wellbeing practitioner for 1 initial session of 60 min (either face-to-face or telephone, dependent on participant preference) followed by up to 10, 30-min sessions over a 12-week period.

*Minimally supported cCBT* consisted of a commercially produced cCBT program (Ocfighter; www.ccbt.co.uk). OCfighter comprises a 9-step CBT approach (focused on exposure and response prevention) to help people with OCD to design, carry out and monitor their own treatment and progress. Participants randomized to OCfighter were given access to the system and were advised to use the program at least 6 times over a 12-week period. Participants received six, 10-min brief scheduled telephone calls from a psychological wellbeing practitioner.

A total of 473 participants were recruited into the trial, most via IAPT services. Three hundred and fifteen participants were randomized to treatments (cCBT = 157; guided self-help = 158) and allocated to one of 93 trained psychological wellbeing practitioners. The remaining participants were allocated to a waiting list for high intensity CBT (control group = 158). Only those participants allocated to one of the two trial interventions (cCBT; guided self-help) were eligible to take part in this qualitative study, as they were able to contribute their views of the trial interventions. Invites were sent out to the first 125 participants allocated to either cCBT or guided self-help); recruitment was discontinued once data saturation was achieved. Forty-four participants returned consent to contact forms; eight of these did not consent to and complete the interview. Reasons included inability to contact participants, refusal to take part due to competing demands and failure to return the consent form.

Thirty-six consented to interview (11 % of all those allocated to an intervention; 18 in each group). More than half were female (57 %); the majority was white-British (94 %) with a mean age of 44 years (range: 22–66 years). The response rate was relatively low (29 % of those invited for interview); there may be several reasons for this, which have been detailed in the strengths and limitations of this study. Interviews took place between October 2012 and January 2014 and were conducted face-to-face in participants’ homes or at the university (46 %) or by telephone (54 %), depending on participant choice and location.

All participants were contacted after the planned completion of the low intensity interventions; however, due to unforeseen delays in allocation to psychological wellbeing practitioners and therapy session bookings, a small number of participants were still undergoing treatment at the time of the interview (*n* = 2), while others had already reached the top of the waiting list and were undergoing or had completed a course of high intensity face-to-face CBT (*n* = 18). Interviews were conducted between 4 and 13 months after participants entered the trial. Participation was voluntary and written informed consent was obtained from all participants prior to the interview.

Interviews were conducted using open-ended, inductive questioning around key topics, outlined in an interview topic guide; this was devised by the research team in collaboration with service users and piloted before use. Interviews were conducted by JK-H, a PhD student in health services research with a background in psychology and a service user researcher with OCD (AF), who was trained prior to conducting the interviews to ensure a mutual understanding of the interview process and focus. The interviews ranged between 27 and 129 min, and were all audio-taped and transcribed verbatim. Participants were offered copies of their transcripts by post for editing and correction purposes, 14 accepted this offer; none requested changes.

The interview data was subjected to a thematic analysis [36], using the constant comparison method [37]. In line with current recommendations [38], data were analyzed without prior knowledge of trial effects. Transcripts were coded and themes identified by JK-H. As themes were identified, they were compared and refined within and across interviews. Disconfirming cases were explored to test the boundaries of the identified themes [39]. Independent verification of emergent themes was achieved through double coding of 50 % of the interview transcripts (PEB; SK). Regular discussion of emergent themes among members of the wider research team ensured that the analysis remained grounded in the data (JK-H; PEB; SK; KL). To preserve anonymity, participants are referred to by a code, containing the participant (P) identifier, gender (F/M) and treatment group allocation (Guided self-help; GSH/cCBT).

## Results

Six major themes emerged from the analysis. Three relate to users’ general experiences with low intensity interventions: i) general expectations of low intensity interventions and the perceived hierarchy of treatment, based on the level of therapist support, ii) engaging with low intensity interventions and the ability of guided self-help to offer an acceptable compromise between accessibility and professional contact and iii) individual differences in the perceived value of therapist support. However, additional themes emerged that appeared unique to the experience of OCD: i) expectations of treatment in OCD, ii) the need for personalization of therapy materials for diverse OCD profiles and levels of knowledge about OCD and its treatment and iii) individual differences in the value of therapist support to facilitate OCD disclosure. Themes are presented below with illustrative quotations; firstly those relating to users’ general perceptions of low intensity interventions, followed by themes specific to users with OCD.

### General perceptions of low intensity interventions

Consistent with previous research on non-OCD samples, we observed individual differences in the acceptability of low intensity interventions. The identified themes are outlined in the following paragraphs.

### General expectations of low intensity interventions and the perceived hierarchy of treatment

Participants perceived a hierarchy of treatments, with high intensity face-to-face treatment as superior and minimally supported self-help (cCBT) as inferior approach. However, participants were pragmatic about trying new treatments, referring to a history of trial and error in finding effective psychological and pharmacological treatments:

*‘I mean, any help is better than none isn’t it? It’s the way I look at it. I mean, we said before about, I was apprehensive about going to see this [inaudible 55:05] but I went because anything is better than nothing. And I was willing to give it a go. So, yes, I would recommend the computer programme, yes.’ (P1008McCBT)*

While high intensity face-to-face therapy remained superior for several participants, the addition of less intensive therapist support in the guided self-help group, even if provided in the form of telephone contact, was perceived to help address some of the limitations of minimally supported cCBT, thus offering a compromise:

*‘So yeah, I do think, yeah, telephone conversation, yeah, I think it would work basically as well, but not as well as one on one, but it's still helpful as well, I would say, talking to somebody on the telephone, yeah.’ (P1443MGSH)*

### Engaging with low intensity interventions

Although participants were generally willing to try out a new treatment, sustaining engagement was a challenge. Experiences of engaging with the low intensity interventions varied between individuals with the same features of self-help considered as positive or negative depending on individual circumstances and preferences. For instance, some participants considered the relative flexibility of accessing self-help materials an advantage both in terms of access and the more informal therapeutic milieu that was created:

*‘Yeah. At the beginning I used to say I’ll do it every Wednesday morning, because Wednesday morning’s the best morning for me to do anything. I’ll go on it. But now I just go on it when I feel like it, and I find that better. I think that’s one of the bonuses of it, you can do it when you want to do it.’ (P1446McCBT)*

Similarly, those who also received telephone support appreciated the relative ease of access to therapy without the barriers associated with face-to-face therapy:

*‘The place I got offered, it was too far … when we found finally, a place that is not far away, and easy travel for me, there wasn’t an appointment in that centre that was suitable for both of us. Plus, the problem was, I have a daughter… I don’t mind, she is a very good girl, and all the time I’m talking to someone she’s usually drawing, but they didn’t let children in the building. The phone… this for me, was easier. I don’t need to go out, still I got the one to one attention… I can’t say it would work for everyone, but personally for me, it was really suitable.’ (P1276FGSH)*

By contrast, other participants felt this flexibility enabled them to avoid therapy or fail to prioritize it, in contrast to face-to-face appointments, which demanded greater commitment. Scheduled therapy sessions were also easier to anticipate and accommodate in users’ daily routines:

*‘I suppose that’s the good thing with the therapy sessions you make an appointment and you go to it so you know you’re going to do it. Whereas when you’re at home you could easily think “Oh I’ll do it tomorrow” and then tomorrow comes and you think “Oh I’ll do it tomorrow,” whereas if you’ve book an appointment and you know you didn’t easily get that appointment you know you’ve got to go to it. I think at home you’ve got that choice of “Oh I’m a bit busy today should I do it tomorrow?”’ (P1027FcCBT)*

As with expectations, the addition of less intensive therapist support was perceived positively as being able to address some of the limitations of cCBT and provide an adequate compromise between the relative benefits of self-help and high intensity face-to-face treatment. Through the comparison of accounts of cCBT and guided self-help, it was possible to identify the two main perceived benefits of the additional therapist support. Firstly, contact with a therapist had a motivating impact and encouraged participants to sustain their engagement. This was perceived to be lacking within the self-help materials alone. Secondly, therapists were perceived to play a valuable role in personalizing therapy. By outlining the relevance of therapy components to their problems and particular circumstances, some users considered the therapist as essential to maximizing the benefit gained from therapy:

*‘It’s really given me, always, good extra help when she said, oh you came a long way, oh well done, you’ve done a really good job. She always encouraged me, even if we didn’t complete one part, what we thought we would do, she always encouraged me to be positive and see these sort of things, and that was the really good part…’ (P1276FGSH)*

*‘I think some of it we talked through and once someone explains to you the diagram (vicious cycle) that relates to your own issues, it clicks, yeah… you need someone to put it in context, because you see the diagram and you just think, well, what’s that got to do with me? What’s that about? Is it modern art?’ (P1156MGSH)*

### The perceived value of therapist support

Individual differences remained a potent factor however, and the less intensive therapist support was insufficient for some participants. For these users, physical and temporal colocation of support was fundamental to their perception of effective treatment, and self-help materials in isolation, even with remote professional support, could not achieve the interpersonal or emotional effects considered necessary:

*‘I mean, really, one thing is, I’m sort in my own environment, and it’s someone that’s phoning from outside that. They’re sort of away from it. Whereas if I’m speaking to you now, I do feel relaxed and what have you.’ (P1207McCBT)*

To some participants the value of therapist support was primarily associated with establishing rapport and the warmth of the client-therapist relationship. These determinants of ‘high quality therapy’ were not satisfied for some users, who considered therapist support in the low intensity interventions as ‘clinical’ in nature:

*‘But she seemed to be working off a script, rather than we have talked together today. And I found it was, you know, not that helpful really, no disrespect to her, she was just doing her job, but she was doing it, in my thought, a clinical way, you know, which I thought anybody could have done really.’ (P1443MGSH)*

### Specific challenges of OCD for low intensity interventions

It was evident that the issues around individual differences in experience and perceptions of the value of therapist support interacted with the unique features of OCD, with important implications for therapy delivery.

### Expectations of treatment in OCD

While the majority of participants were willing to try the low intensity interventions (though with some reservations and low expectations), some expressed specific reservations relating to ambivalence around their symptoms. Although a burden to many, some participants valued certain aspects of OCD, and some feared change sufficiently to lessen their motivation to initiate psychotherapy, regardless of its delivery format:

*‘However, on the plus side, having OCD was been very beneficial because I’ve achieved lots in my life which I probably wouldn’t have done without the perfectionist of the OCD and that sort of thing. So it’s like the right tool in the right hands, I love it. If I was to pick something I wouldn’t say I wouldn’t want it because it’s been better than it has been worse…’ (P1213MGSH)*

Prior research has discussed the effect of users’ negative self-concept on OCD onset and progression [40]; however, some participants considered certain OCD symptoms as integral and valued parts of their self-concept:

*‘Because my biggest worry when I first went, and I did tell him, was I going to go from being a clean freak to being slovenly really. He said no, you will never alter… because I was worried about that, and he said you will never alter the actual person that you are, you’ll just learn how to deal with things so you don’t need to over-clean all the time and overdo things.’ (P1263FGSH)*

Such reservations over change may not be unique to low intensity interventions. However, user dropout may be particularly high in low intensity interventions with minimal therapist contact. Users’ uncertainty over the ‘new self’ after treatment may require therapist support in affirming users and outlining the role and boundaries of therapy and its impact, demonstrating how expectations unique to OCD must be addressed to support uptake and engagement in this user group.

### Personalization of materials

As discussed previously, self-help materials were commonly considered too generic and many users struggled to engage with content that was less relevant for them. This need for personalization of therapy materials was especially prominent for this sample given their varied symptoms, compared to more homogenous disorders. The relevance of therapy materials was critical to treatment continuation in both cCBT and guided self-help and was considered a key benefit of contact with a professional who could individualize the materials and support application of the techniques to the participants’ specific circumstances. Without this personalization, participants were likely to disengage:

*‘And I think maybe if the programme was to work any better, I think it might be more optional, you know, more going in depth to someone's particular OCD because, you know, there's hundreds of different types of OCD. And I don't think a generic programme can just, you know…maybe can help everybody. It might help, you know… the intentions obviously there are good, but I don't think in reality… in reality, I felt it a bit, you know, this is just too generic. But I equally understand that it's very difficult to write something so specialised for everybody on the same site… so consequently, I think I just stopped using it after a while and just thought I'm not getting anything out of this.’ (P1188McCBT)*

Aside from participants’ specific OCD symptoms, their level of insight into and expertise relating to OCD affected the relevance of low intensity interventions. While there were participants in both groups (cCBT; guided self-help) who considered the interventions too ‘basic’, this criticism was particularly prominent in minimally supported cCBT, where the potential for personalization was limited through a lack of therapist support:

*‘…and it wasn’t a, sort of, acute thing, it’s a chronic thing that’s been going on in the background for however long. I think maybe somebody who had… who hasn’t had that opportunity, that awareness, that exposure to that many different things, and books, and ideas, they might never have heard of CBT. They might not know that there’s a link between your thoughts and your actions, so all of that would be new information for them, and therefore it might work for them, it might be useful just having that first introduction.’ (P1243FcCBT)*

Guided self-help, containing more intensive therapist support and hence, a greater potential for personalization and adaptation in line with users’ needs and existing level of knowledge about OCD and its treatment may offer a useful compromise:

*‘The book wasn’t completely useless, but if I’d have just had the book without the therapist I don’t think I would have made the improvements that I did do…when I talked about it with him, even thought it was basically common sense what he was telling me, because I’d never thought about it because that was my life the way it was, it was helpful the fact that he was putting everything into context for me.’ (P1263FGSH)*

### Individual differences in the perceived value of therapist support for OCD disclosure

While both previous themes indicated the value of professional support to overcome concerns about the impact of treatment or to personalize materials, individual variation was still evident. This became apparent regarding the benefits or limitations of working with materials alone or remotely in terms of users’ anxiety around disclosure of their symptoms. It is recognized in OCD that the experience of egodystonic symptoms (e.g., obsessions of a violent, religious or sexual nature) is commonly associated with shame and embarrassment and consequently, disclosure is often delayed [27, 41]. For some participants, technology-based interventions such as cCBT and guided self-help offered a way of accessing therapy without the need for face-to-face contact, preserving anonymity or providing a valuable distance:

*‘What I really did like is the fact that… I didn’t have to tell anybody else face to face. It felt like it was a way of coping privately but in a structured way. And I found that a real relief to be honest.’ (1167FcCBT)*

*‘I didn’t go to the bits that concerned me (violent obsessions) because I didn’t…and I haven’t yet with anybody apart from you, funnily enough… I find it easier because you’re on the phone, so I’m not looking at you eye to eye.’ (P1213MGSH)*

This enhanced ‘privacy’ was discussed by users both in terms of the environment within which therapy was received (commonly participants’ own home) and in terms of the program delivering treatment. A secure cCBT platform enabled users to disclose sensitive information by preserving their confidentiality and could in fact allow a more authentic engagement with therapy:

*‘Because it was passworded as well it became private and I knew that nobody would be looking at it… I felt like it was mine and mine only, like I could be really honest because no one was looking at it. There are some questions on there that I've answered really honestly, but I don't think I would've answered them to a person and some of them I struggled answering with x (researcher). So I think from that you'd probably get a better result because it's private and you're not telling anyone anything really; you're just doing it with yourself so you can be more honest.’ (P1198FcCBT)*

This is in contrast to users who considered the face-to-face therapeutic encounter as necessary to overcome the shame associated with OCD or to break the cycle of avoidance. Following the direct experience of face-to-face therapy, the observed differences hint at a possible disjointedness between users’ initial treatment preferences and subsequent service satisfaction and may thus represent a change in users’ opinion over time rather than differences between individual users:

*‘I think, probably face to face is necessary, I think, you have to actually get over that shame, if possible.’ (P1006FcCBT)*

*‘The phone was okay, but when you go in face to face, the person can tell whether you’re, you know, you’re just trying to skip the question, or you haven’t done as they’ve said… or say what they want you to say, because sometimes you think, oh, I should have done that, I haven’t, but they know…’ (P1282MGSH)*

## Discussion

The aim of this study was to explore user perspectives on two low intensity CBT interventions. We aimed to explore differing perceptions of the need for therapist support and whether OCD had a unique impact. We identified a broad continuum of therapy acceptability, placing face-to-face therapy and technology-based interventions (cCBT) at two opposing ends. Research evidence supports the importance of the therapeutic relationship in promoting engagement with low intensity interventions [42]. The importance of the therapist emerged as a central thread throughout the data, with the expectation of many users focusing on the interpersonal aspects as key components of ‘good quality’ care. This meant that guided self-help, which combined the accessibility of self-help materials with support from a professional, was considered an acceptable compromise, consistent with other research [43]. The data enabled us to identify what factors impacted on the acceptability of this compromise. Specifically, participants who required therapist support for external encouragement and personalization of therapy found the level of support provided in guided self-help acceptable. By contrast, those users who valued the interpersonal and physical nature of a face-to-face relationship considered low intensity interventions far less acceptable.

A relative lack of robust empirical evidence in favor of the importance of the interpersonal aspects over specific evidence-based therapy mechanisms means that guidelines will likely continue to focus on content over context in the delivery of CBT [44]. The data presented here demonstrate however that preferences for mode of delivery and level of support impact directly on therapy engagement and influence perceptions of the therapeutic value of a particular therapy modality. As an apparent consequence of the level of therapist contact offered as part of the two respective interventions, guided self-help saw higher rates of engagement compared to cCBT (58 % vs. 35 % self-reported engagement). By offering an ever-present health-technology (self-help manual), combined with regular, structured support, guided self-help retained the advantages of low intensity interventions (facilitating disclosure; accommodating competing responsibilities), without adopting the limitations of either minimally supported- or high intensity therapy, thus maximizing its acceptability across a broader proportion of the OCD population.

Users’ criticisms of cCBT focused predominantly on its inflexible content and limited ability for tailoring. The therapist’s role in maximizing responsiveness and flexibility may be particularly important in OCD. Professional support may also help overcome barriers for people with OCD who are ambivalent about change. This is reminiscent of research on computerized therapies for depression, which increasingly focuses on managing initial user expectations and facilitating acceptance of novel delivery formats [45], and identifies how expectations unique to OCD must be considered in any similar work.

Consistent with previous research, there was individual variation in whether lower levels of contact were perceived as adequate, or whether distance and privacy were considered more valuable. This demonstrates the need for flexibility in the delivery of low intensity interventions, responsive not only to clinical factors but also to user need, experience and preferences. Furthermore, there is a need to recognize the impact of specific features of OCD. The relative value of different delivery formats may vary with symptom profile or the level of symptom-related shame.

### Implications

Our findings have important implications for the development of low intensity interventions and their integration into existing OCD services. Due to varied symptom patterns, many users found the therapeutic content lacking in personal relevance. In addition, the therapy materials alone failed to accommodate users’ varied levels of prior knowledge about OCD and its treatment. Combined with a lack of therapist contact to personalize treatment, technology-delivered therapy may not be appropriate to many individuals with OCD. Similarly, to those with prior knowledge of OCD and its treatment, cCBT was considered lacking in depth and potential for personalization. Nevertheless, as shame and stigma are barriers to seeking treatment for OCD [30], low intensity interventions may overcome some barriers to accessing face-to-face therapy. Those experiencing significant shame may be more likely to value and benefit from cCBT, retaining privacy and confidentiality. Conversely, the ambivalence over change and fear over the ‘new self’ after treatment, as experienced by some users, may require more intensive therapist support to be adequately addressed and resolved. While discussion of ‘stepping up’ to more intensive therapies in the stepped care model typically focuses on clinical factors, our findings demonstrate that preferences for delivery format should also be considered. It is also important to note that users could not always reliably anticipate what format of delivery was going to be effective for them and acceptability may change following exposure to new treatment methods.

It has been demonstrated that ‘responsive regulation’ in mental health services, whereby the number of therapy sessions received is decided on an individual basis in response to progress rather than adhering to a pre-defined protocol, may be effective and efficient [46]. It may be that similar flexibility in the intensity- and mode of professional support for self-help treatments could also maintain efficiencies, while being responsive to individual preferences. However, although protocols combining self-help materials with therapist support are more acceptable to service users [47], it is unclear how such protocols should best be implemented in practice, particularly in regard to maintaining the resource gains associated with lower levels of professional support [48]. Future research should explore how services can best provide additional support in an efficient and acceptable way.

### Strengths and limitations

The strengths and limitations of this study ought to be considered when interpreting the present findings. This study offers novel and important insights into the individual differences, which drive uptake, engagement and acceptability of low intensity CBT interventions in adults with OCD, a condition with a limited evidence-base. This study includes two guideline-recommended interventions (guided self-help/cCBT), a key strength in terms of its relevance to mental health service delivery more broadly. However, common limitations of qualitative research around the generalizability of the findings also apply to the present study. The response rate was low, with only 29 % of those invited, participating in the interview. Hence, the study sample may represent a biased subgroup of those eligible for participation. There may be several reasons for this: Participation in the trial involved four assessments, lasting 1-3 h each, and the prospect of another interview may have felt burdensome. Moreover, those participants who had completed the quantitative assessments may have discarded the postal invitation, no longer considering themselves active participants of the trial. Finally, the lack of remuneration may account in part for the low response rate. As study participants represent a treatment-seeking sample, the present findings may be biased in terms of user motivation for treatment. Furthermore, while participants were recruited from across the UK, ethnic minorities were under-represented. The views of a more diverse sample are needed to inform culturally relevant care. Recruiting participants from waiting lists for high intensity face-to-face therapy may have lessened users’ perceived need and motivation to engage with low intensity resources. However, the present circumstances more closely reflect the reality of mental health service delivery within the UK where high intensity services become available if low intensity first-line treatment proves ineffective.

## Conclusions

The present findings provide useful insights to inform the future of low intensity interventions within UK mental health services for OCD. While there may be other factors affecting therapy initiation, continuation and acceptability, the implications of which are most adequately determined through clinical judgment these findings offer important guidance on issues impacting on therapy engagement and experience. Low intensity interventions may indeed have a place in mental health services if the aim is to increase choice and accommodate the needs of all users at different stages of the treatment journey. The integration of low intensity interventions and the decision-making process involved in matching users to adequate psychological therapies however may require revision. The challenge now is in balancing therapy acceptability for users with service efficiency.

### Ethics, consent and permissions

Ethical approval was granted by a local research ethics committee (National Research Ethics Service [NRES] Committee North West – Lancaster; 11/NW/0276).

All participants gave written informed consent to participate before the interview. All participants consented for anonymized quotes to be used in the write-up of the study findings.

### Availability of data and materials

Data will not be shared, as ethical approval has only been granted for the use of anonymized quotes in the write-up of the interview data.

## Abbreviations

CBT: cognitive behavioral therapy
cCBT: computerized cognitive behavioral therapy
GSH: guided self-help
IAPT: Increasing Access to Psychological Therapies
OCD: obsessive-compulsive disorder
OCTET: Obsessive-Compulsive Treatment Efficacy Trial
RCT: randomized controlled trial
Y-BOCS: Yale-Brown Obsessive Compulsive Scale
